# Rethinking Onconephrology: A Nephro-Nutritional Integrated Approach in Patients with Chronic Kidney Disease and Urological Malignancies

**DOI:** 10.3390/nu18121863

**Published:** 2026-06-09

**Authors:** Francesco Trevisani, Andrea Angioi, Agnese Monti, Michela Passera, Fabiana Selvaggi, Matteo Floris, Andrea Salonia, Francesco Montorsi, Umberto Capitanio, Arianna Bettiga

**Affiliations:** 1 Division of Experimental Oncology, Department of Urology, URI—Urological Research Institute, IRCCS San Raffaele Hospital, 20132 Milan, Italy; 2 Department of Urology, IRCCS San Raffaele Hospital, 20132 Milan, Italy; salonia.andrea@hsr.it (A.S.); montorsi.francesco@hsr.it (F.M.); capitanio.umberto@hsr.it (U.C.); bettiga.arianna@hsr.it (A.B.); 3 Department of Nephrology, Dialysis, and Transplantation, ARNAS G. Brotzu, 09047 Cagliari, Italy; andrea.angioi@aob.it (A.A.); matteo.floris@aob.it (M.F.); 4 URI—Urological Research Institute, IRCCS San Raffaele Hospital, 20132 Milan, Italy; agnesemonti02@gmail.com; 5 Department of Biosciences, University of Milan, 20133 Milan, Italy; michela.passera2000@gmail.com (M.P.); fabiana.selvaggi@gmail.com (F.S.)

**Keywords:** onconephrology, CKD, MCPD, BIA

## Abstract

**Background**: Nutritional therapy is central in the management of chronic kidney disease (CKD) and cancer, yet these conditions impose partially conflicting requirements. The 2024 KDIGO guideline recommends a controlled protein intake (~0.8 g/kg/day) to reduce metabolic burden in non-dialysis CKD patients, whereas the ESPEN (European Society for Clinical Nutrition and Metabolism) guidelines support higher protein intake (≥1.0–1.5 g/kg/day) to prevent cancer-related malnutrition. Evidence guiding patients affected by both conditions is limited. We evaluated the effects of a Mediterranean-like controlled protein diet in onconephrological patients compared with CKD controls. **Methods**: In this retrospective study, 358 CKD patients (183 onconephrological, 175 controls) were followed at a tertiary center (2017–2024). Patients received a protein-controlled diet (0.6–1.0 g/kg/day) tailored to comorbidities and nutritional status. Nutritional assessment included bioelectrical impedance analysis and anthropometry. Renal function was evaluated using creatinine and cystatin C, and measured GFR by iohexol clearance at baseline and 12 months. **Results**: Baseline body composition was comparable between groups. After intervention, serum urea significantly decreased in both groups, without a decline in measured or estimated GFR. Fat mass and central adiposity indices were reduced, while lean mass and phase angle remained stable. No evidence of protein–energy wasting or catabolic activation emerged. Longitudinal analyses showed no significant time × cancer interaction for renal function or most bioimpedance-derived body composition parameters. However, at extended follow-up, arm circumference and tricipital skinfold thickness showed significant time × cancer interactions, suggesting different longer-term peripheral anthropometric trajectories according to cancer status. **Conclusions**: In this retrospective real-world cohort, structured nephro-nutritional management with an individualized Mediterranean-like controlled protein prescription was associated with preserved renal function and no evidence of overt nutritional deterioration in onconephrological patients. These findings support the feasibility and apparent safety of this approach in selected patients, while highlighting the need for prospective studies with objective dietary adherence assessment and longer-term evaluation of cancer-related anthropometric trajectories.

## 1. Introduction

The growing intersection between chronic kidney disease (CKD) and cancer represents an increasingly relevant clinical challenge [1]. These conditions frequently coexist and interact bidirectionally: oncologic treatments, including surgery, chemotherapy, immunotherapy, and targeted therapies, may impair kidney function, while pre-existing or treatment-induced renal dysfunction can limit therapeutic options and worsen outcomes [2,3]. In this setting, nutritional management is central to preventing metabolic derangements and functional decline, with the aim of preserving muscle mass and limiting malnutrition, a frequent complication in cancer associated with increased morbidity and mortality [4,5]. Nutrition is therefore a key component of care in both CKD and oncology, particularly within a multidisciplinary framework. Dietary interventions have been investigated as a strategy to slow CKD progression toward end-stage renal disease (ESRD). Low-protein diets (LPDs), although historically limited by concerns regarding nutritional status, have been shown to improve metabolic control and delay the need for renal replacement therapy (RRT), supporting their use in selected patients [6,7,8]. A Mediterranean-style low-protein diet (MCPD), typically providing 0.6–0.8 g/kg/day of protein, is associated with reductions in uremic toxins, improved acid–base and electrolyte balance, and attenuation of inflammation and oxidative stress. These effects translate into improvements in blood pressure, endothelial function, and metabolic profile, as well as reduced proteinuria and slower decline in kidney function [9,10,11]. Additional data support a reduction in markers of tubular injury and interstitial fibrosis, reinforcing the nephroprotective role of protein restriction. When individualized, LPDs are associated with slower CKD progression. Recommendations on protein intake in CKD have evolved over time. The 2020 KDIGO guidelines supported more restrictive targets (≈0.55–0.6 g/kg/day) in non-dialysis CKD, whereas the 2024 KDIGO guideline suggests a protein intake of approximately 0.8 g/kg/day, with emphasis on individualization and avoidance of protein–energy wasting. This change reflects the limited strength of available evidence and ongoing uncertainty regarding the optimal degree of protein restriction. By contrast, ESPEN guidelines for cancer recommend higher protein intakes (1.0–1.5 g/kg/day, up to 2.0 g/kg/day in highly catabolic states) [12,13]. Although appropriate in the context of cancer-related catabolism, such targets may not be optimal in patients with concomitant CKD, in whom high protein intake can increase intraglomerular pressure and hyperfiltration, potentially accelerating renal decline [14]. Systemic inflammation further alters macronutrient metabolism, supporting the need for tailored nutritional strategies. Among hypoproteic approaches, the Mediterranean diet represents a structured and clinically applicable model. The MCPD combines controlled protein intake with a high consumption of plant-based foods, fiber, antioxidants, and unsaturated fats, while limiting sodium, refined sugars, and nitrogen-rich animal protein sources [15,16,17,18]. This composition supports renal function while maintaining body composition through adequate energy intake and high nutritional quality. Previous studies have shown that MCPD improves metabolic and clinical parameters in CKD, promotes weight stability, reduces inflammation, and lowers the risk of protein–energy wasting, including in patients with cancer. Its palatability and long-term sustainability may also favor adherence and quality of life [19,20,21].

**Figure 1:**
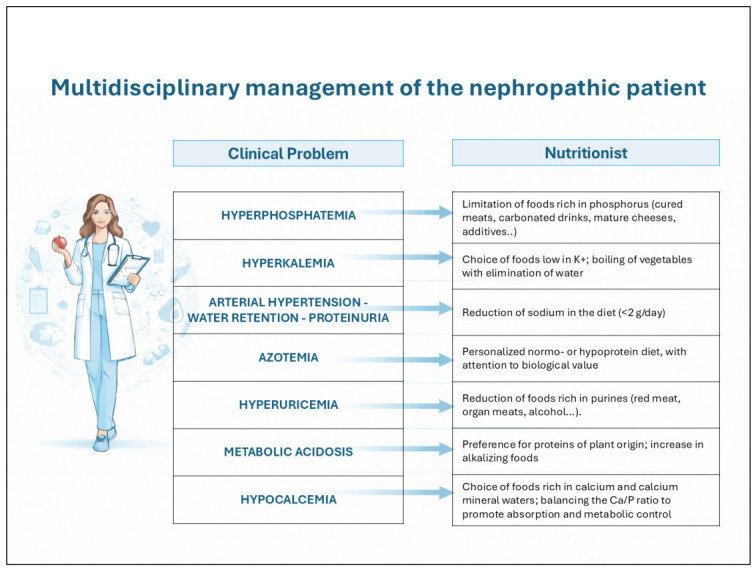
Therapeutic objectives of nutritional diet therapy.

Nutritional therapy is a fundamental point in the treatment of kidney disease and must be perfectly integrated with the pharmacological therapy managed by the nephrology specialist. Nutritional therapy and pharmacological therapy work in concert on the treatment of the main symptoms and signs of CKD (hypertension, anemia, acidosis, calcium–phosphorus metabolism imbalances, etc.), enhancing their effects (Figure 1).

In this study, we evaluated the impact of a Mediterranean Controlled Protein Diet (MCPD) in a consecutive cohort of 183 onconephrological patients, compared with 175 non-oncological CKD controls. Using a multidisciplinary approach integrating nephrology and clinical nutrition, we assessed clinical outcomes, body composition, and preservation of residual renal function.

## 2. Materials and Methods

An observational retrospective study was conducted on a cohort of 358 patients followed at a tertiary care research institute (IRCCS San Raffaele Hospital, Milan, Italy) between 2017 and 2024. The study population was stratified into onconephrological patients (ON, n = 183), considered as cases, and non-oncological CKD patients (NTN, n = 175), serving as controls. This design allowed assessment of the applicability and safety of nephrology-oriented dietary strategies in patients with concomitant malignancy.

All patients underwent standardized nephrological and nutritional evaluations at baseline and after 12 and 18 months (±3 months). A Mediterranean-like controlled protein diet (MCPD) was prescribed and individualized according to CKD stage, measured glomerular filtration rate (GFR), comorbidities, and overall clinical status.

The dietary protocol reflected clinical practice at the time of patient inclusion, when more restrictive protein targets were commonly adopted. Accordingly, protein intake ranged from 0.6 g/kg/day in patients with stages G3–G5 CKD and stable nutritional status, with adjustments up to 0.8–1.0 g/kg/day based on individual clinical requirements and treatment goals.

Total energy intake was set at 25–35 kcal/kg/day to maintain neutral nitrogen balance and preserve nutritional status. Macronutrient distribution included at least 30% of total energy from lipids and 55–60% from carbohydrates. When required, protein-free commercial products were used to ensure adequate caloric intake. The diet was characterized by a high intake of plant-based foods, including 500–700 g/day of fruits and vegetables, with emphasis on fiber and unsaturated fats, consistent with a Mediterranean dietary pattern.

Electrolyte intake was individualized according to clinical status, with general targets of sodium < 100 mmol/day, calcium 800–1000 mg/day, phosphorus ~800 mg/day, and potassium 2–4 g/day. Micronutrient supplementation (e.g., vitamin D, calcium, folic acid, and vitamin B12) was prescribed when clinically indicated.

Laboratory parameters were collected at baseline and follow-up, including serum creatinine, cystatin C, and urea. Estimated GFR was calculated using the CKD-EPI 2021 equation, and measured GFR was obtained using iohexol plasma clearance. CKD staging was assigned according to KDIGO 2024 criteria. Measured GFR was used to minimize bias related to sarcopenia and systemic inflammation.

Nutritional status was assessed at each time point through clinical evaluation, body weight, presence of edema, and bioimpedance analysis, with specific attention to body cell mass and phase angle.

Exclusion criteria were: age < 18 years, eGFR ≥ 90 mL/min/1.73 m^2^, unstable renal function within 3 months, end-stage renal disease requiring dialysis, ongoing chemotherapy or immunotherapy, metastatic disease, Nutrition Risk Screening (NRS-2002) score < 3, and lack of informed consent.

Clinical data included comorbidities (hypertension, diabetes mellitus, and solitary kidney) and cancer diagnoses (urothelial carcinoma, renal cell carcinoma, prostate cancer, and other urological malignancies).

The study was approved by the Institutional Ethics Committee (San Raffaele Hospital, Milan; Protocol URBBAN, approved 3 March 2014), and all patients provided written informed consent.

### 2.1. Assessment of Nutritional Status

Anthropometric measurements were performed to assess each patient’s nutritional status. Body weight (BMI, measured to the nearest 0.1 kg) and height (measured to the nearest 0.1 cm) were collected, and BMI was calculated by dividing weight (kg) by height squared (m^2^). Waist circumference (WC, measured to the nearest 0.1 cm) was collected midway between the inferior margin of the last rib and the crest of the ilium in a horizontal plane. The hip circumference (HC) is taken around the widest part of the buttocks, typically by standing with your feet together and keeping them parallel to the ground. Waist-to-hip ratio (WHR) was calculated by WC (cm) divided by HC (cm). Waist-to-height ratio (WHtR) was calculated by WC (cm) divided by height (cm). Wrist-to-height ratio is a method to estimate body frame size (small, medium, or large), and it was calculated by wrist circumference (cm) divided by height (cm); for women, a ratio (height/wrist in cm) above 11.0 indicates a small frame, 10.1–11.0 is medium, and below 10.1 is large. For men, a ratio above 10.4 is small, 9.6–10.4 is medium, and below 9.6 is large.

Bioelectrical impedance analysis (BIA-Dex^®^ Mascaretti srl-sinusoidal 50 kHz waveform current, intensity of 0.8 A) was used to analyze body composition. Data were analyzed through the software BIA-Dex CLOUD Plus. We collected data for phase angle (PA), body cellular mass (BCM%), body cellular mass/height^2^ ratio (BCMI), fat mass (FM%), fat-free mass (FFM%), extracellular mass/BCM ratio (ECM/BCM), extracellular water/intracellular water ratio (ECW/ICW), total water (TBW%), fat mass/height^2^ ratio (FMI), and fat-free mass/height^2^ ratio (FFMI). The validated NRS-2002 was used to detect malnutrition for nephrological patients as recommended by the ESPEN-recommended tool [22].

### 2.2. Statistical Analysis

Statistical analyses were performed using SPSS statistical software, version 31. Continuous variables are reported as median and range, while categorical variables are reported as number and percentage. Between-group comparisons at baseline were performed using the Mann–Whitney U test for continuous variables and the χ^2^ test or Fisher’s exact test for categorical variables, as appropriate.

Longitudinal within-group changes from baseline to follow-up were assessed using paired Wilcoxon signed-rank tests and were considered exploratory descriptive analyses. These tests were not used to infer between-group similarity or difference in longitudinal trajectories.

To evaluate whether cancer status modified changes over time, mixed-design repeated-measures ANOVA models were used, including time as the within-subject factor and cancer status as the between-subject factor. The time × cancer interaction was considered the key statistical term for assessing differential longitudinal trajectories between onconephrological and non-oncological CKD patients. Partial eta squared (η^2^) was reported as a measure of effect size. A two-sided *p*-value < 0.05 was considered statistically significant. Given the retrospective design and the number of outcomes examined, analyses were interpreted as exploratory and hypothesis-generating. Analyses were performed using SPSS statistical software, version 31.

## 3. Results

### 3.1. Clinical and Demographic Findings of the Patients

A total of 358 patients with chronic kidney disease (CKD) were included in the analysis. The cohort comprised 183 onconephrological patients (ON, 51.1%), defined by a history of urological malignancy within the preceding 12 months and considered as the case group, and 175 patients with CKD without malignancy (NTN, 48.9%), who served as controls. CKD was defined as an estimated glomerular filtration rate (eGFR) < 90 mL/min/1.73 m^2^ according to KDIGO 2024 classification. Baseline demographic and clinical characteristics of the overall cohort and according to group stratification are summarized in Table 1.

The median age of the study population was 67 years (range 16–86). Patients in the ON group were significantly older than those in the NTN group, although the absolute difference was modest (69 vs. 65 years; *p* = 0.031). Male sex predominated in both groups, with a significantly higher proportion among ON patients compared with NTN patients (83% vs. 68.6%, *p* = 0.002).

Median body mass index (BMI) was similar between groups (26.05 vs. 26.20 kg/m^2^, *p* = 0.991), and no differences were observed in BMI category distribution (underweight, normal weight, overweight, and obesity). Overall, overweight and obesity were highly prevalent in both groups, which is consistent with the metabolic profile typically observed in CKD populations. Wrist circumference/height ratio has been suggested to be an easy and well-known index to estimate skeletal frame size, which aids in assessing appropriate body weight, and, alongside BMI [23], some studies also supported the association between adiposity and wrist circumference [24].

Our population was characterized by a large–medium frame; only 11.2% had an Ectomorph phenotype.

CKD stage distribution differed between groups (*p* = 0.005), with a predominance of stage IIIa–IIIb in both cohorts. The prevalence of diabetes mellitus and hypertension was comparable between groups. In contrast, single-kidney status was significantly more frequent among ON patients than NTN patients (47% vs. 13.1%, *p* = 0.001), reflecting prior nephrectomy in a substantial proportion of patients with oncological disease.

Among onconephrological patients, kidney cancer was the most frequent malignancy (46%), followed by urothelial carcinoma (32%) and prostate cancer (14%), whereas non-urological malignancies accounted for a minority of cases (8%) (Table 2).

### 3.2. Baseline Anthropometric Parameters and Nephrological Assessment of the Patients

At baseline, no statistically significant differences were observed between ON and NTN patients across the majority of BIA-derived parameters (Table 2). Indices of muscle mass and cellularity, including body cell mass index (BCMI), BCM percentage, fat-free mass index (FFMI), fat-free mass percentage, skeletal muscle index, and phase angle, were comparable between groups. Similarly, parameters reflecting hydration status and extracellular compartment distribution (ECM/BCM ratio, ECW/ICW ratio, and total body water percentage) did not differ significantly.

Measures of adiposity, including fat mass index (FMI) and fat mass percentage, as well as anthropometric indicators of central adiposity (waist circumference, waist-to-hip ratio, and waist-to-height ratio), were also similar between ON and NTN patients. Body frame index (Grant morphotype) did not show significant between-group differences.

Overall, baseline nutritional status and body composition appeared well-balanced and comparable between oncological and non-oncological CKD patients.

**Table 2:** Baseline anthropometric parameters and body composition were assessed by bioelectrical impedance analysis. Data are presented as median (minimum–maximum). Comparisons between oncological and nephropathic patients were performed using the Mann–Whitney U test. A two-sided *p*-value < 0.05 was considered statistically significant.

	Median (Min–Max)	Stratified Median (Min–Max)	*p*-Value
BCMI (kg/m^2^)	11.52 (6.38–17.54)	ON 11.40 (6.72–17.54)	0.698
		NTN 11.48 (6.38–17.08)	
BCM (%)	44.1 (18.4–57.7)	ON 44.45 (25.7–57.7)	0.63
		NTN 44.1 (18.4–57.0)	
ECM/BCM	0.84 (0.61–1.6)	ON 0.85 (0.61–1.29)	0.746
		NTN 0.84 (0.63–1.60)	
FMI (kg/m^2^)	5.39 (1.3–21.7)	ON 4.70 (1.3–18.8)	0.668
		NTN 4.70 (1.4–21.7)	
FM (%)	17.7 (7.0–54.5)	ON 17.60 (7.0–47.7)	0.657
		NTN 17.75 (7.0–54.5)	
FFMI (kg/m^2^)	21.2 (14–35.8)	ON 21.20 (14.0–28.6)	0.598
		NTN 21.20 (14.60–35.80)	
FFM (%)	82.4 (45.5–93.0)	ON 82.25 (45.5–93.0)	0.665
		NTN 82.4 (52.6–93.0)	
SMI (kg/m^2^)	11.1 (6.2–17.2)	ON 11.2 (6.2–15.2)	0.821
		NTN 11.1 (6.7–17.2)	
PA (°)	6.2 (3.3–9.4)	ON 6.10 (3.9–8.9)	0.319
		NTN 6.20 (3.3–9.4)	
TBW (%)	59.4 (32.8–67.1)	ON 59.5 (38.0–67.1)	0.572
		NTN 59.35 (32.8–67.1)	
ECW/ICW	0.84 (0.55–1.96)	ON 0.84 (0.57–1.50)	0.439
		NTN 0.84 (0.55–1.96)	
Waist circumference (cm)	94.0 (64–136)	ON 94 (70–136)	0.865
		NTN 94.5 (64–128)	
Hip circumference (cm)	101.0 (81–139)	ON 101 (89–139)	0.573
		NTN 100 (81–127)	
WHR	0.938 (0.68–1.30)	ON 0.936 (0.68–1.30)	0.552
		NTN 0.941 (0.69–1.14)	
WHtR	0.547 (0.39–1.09)	ON 0.545 (0.43–1.09)	0.841
		NTN 0.549 (0.39–0.84)	
BMI (kg/m^2^)	26.15 (16.69–45.23)	ON 26.05 (16.69–43.21)	0.991
		NTN 26.20 (16.88–45.23)	

Baseline nephrological assessment showed higher serum creatinine and cystatin C levels in ON patients, together with lower measured glomerular filtration rate (mGFR assessed by iohexol clearance) and lower estimated GFR values calculated using both creatinine- and cystatin C-based CKD-EPI equations, compared with NTN patients (Table 3). These differences were statistically significant but remained within the expected range for the corresponding CKD stages.

Serum urea levels were modestly higher in ON patients compared with NTN patients (*p* = 0.043). Despite these between-group differences, renal function parameters were clinically stable at baseline in both groups, supporting a reliable longitudinal evaluation of renal and nutritional outcomes following dietary intervention.

### 3.3. Longitudinal Changes in Body Composition and Anthropometric Parameters

In the overall study population, longitudinal analyses comparing baseline and follow-up values showed significant changes in several anthropometric and body composition parameters (Table 4).

Based on BMI classification, 61.4% of patients were overweight or obese at baseline (Table 1). At follow-up, BMI decreased significantly in both groups (ON: 26.05 [16.69–43.21] to 25.75 [17.96–36.04] kg/m^2^, *p* = 0.003; NTN: 26.20 [16.88–45.23] to 25.0 [10.38–38.95] kg/m^2^, *p* = 0.008).

Anthropometric measures of central adiposity decreased over time. Waist circumference was reduced in both ON (94 [70–136] to 93 [74.5–120.0] cm; *p* = 0.002) and NTN patients (94.5 [64–128] to 91 [66.0–113.0] cm; p = 0.010). WHR decreased significantly in ON patients (0.936 [0.68–1.30] to 0.927 [0.49–1.13]; *p* = 0.014), whereas no significant change was observed in NTN patients (0.941 [0.69–1.14] to 0.932 [0.72–1.10]; *p* = 0.249). WHtR decreased in both groups (ON: 0.545 [0.43–1.09] to 0.541 [0.42–0.74], *p* = 0.002; NTN: 0.549 [0.39–0.84] to 0.532 [0.36–0.70], *p* = 0.010).

Regarding body composition, fat mass index (FMI) decreased significantly in ON patients (*p* = 0.014), while no significant change was observed in NTN patients. Fat mass percentage showed a trend toward reduction in ON patients (*p* = 0.053) and remained unchanged in NTN patients (*p* = 0.232).

Lean tissue parameters, including fat-free mass index (FFMI), fat-free mass percentage, body cell mass (BCM and BCMI), and skeletal muscle index, did not change significantly over time in either group. Phase angle remained stable in both ON and NTN patients throughout follow-up.

Hydration-related parameters, including total body water and ECW/ICW ratio, did not show significant changes between baseline and follow-up in either group.

Hip circumference remained stable in ON patients and decreased in NTN patients (*p* < 0.001). Overall, no worsening was observed in body composition or anthropometric parameters over time in either group.

At extended follow-up (T0–T2), significant changes in body composition parameters were observed in both groups (Table 5).

In the ON group, body cell mass percentage (BCM%) increased significantly (44.45 to 46.3; *p* = 0.025), while fat mass percentage (FM%) decreased (17.60 to 17.15; *p* = 0.013). Fat-free mass percentage (FFM%) increased (*p* < 0.001), and total body water also showed a significant increase (*p* = 0.014). Hip circumference decreased (*p* = 0.014). No significant changes were observed in BCMI, fat mass index, skeletal muscle index, phase angle, ECW/ICW ratio, or waist-related indices.

In the NTN group, fat mass index (FMI) decreased significantly (*p* = 0.012), along with fat mass percentage (17.75 to 14.0; *p* = 0.018). Fat-free mass percentage increased (*p* < 0.001), and total body water increased (*p* = 0.016). No significant changes were observed in BCM parameters, skeletal muscle index, phase angle, hydration ratios, or waist-related measures.

Overall, most changes over time involved adiposity-related parameters and body composition distribution, while muscle mass indices and phase angle remained stable.

### 3.4. Longitudinal Nephrological Outcome Improvements

No significant changes in renal function parameters were observed between baseline and follow-up in either group (Table 6).

In the ON group, serum creatinine remained stable (1.54 to 1.57 mg/dL; *p* = 0.18), as did cystatin C (1.34 to 1.35 mg/L; *p* = 0.43), measured GFR (42.35 to 41.0 mL/min/1.73 m^2^; *p* = 0.097), and estimated GFR values derived from creatinine (*p* = 0.48), cystatin C (*p* = 0.28), and combined equations (*p* = 0.813).

Similarly, in the NTN group, no significant changes were observed in creatinine (1.40 to 1.64 mg/dL; *p* = 0.57), cystatin C (1.22 to 1.36 mg/L; *p* = 0.75), measured GFR (50.70 to 39.15 mL/min/1.73 m^2^; *p* = 0.46), or estimated GFR values (all *p* > 0.05).

However, serum urea levels decreased significantly in both groups (ON: 55 to 49 mg/dL; *p* < 0.001; NTN: 50 to 47 mg/dL; *p* = 0.01), and the proportion of patients with urea values > 50 mg/dL also decreased in both groups (*p* < 0.001).

At extended follow-up, renal function parameters remained stable in both groups.

In the ON group, no significant differences were observed in serum creatinine (1.54 to 1.73 mg/dL; *p* = 0.676), cystatin C (1.34 to 1.46 mg/L; *p* = 0.540), measured GFR (42.35 to 37.9 mL/min/1.73 m^2^; *p* = 0.519), or estimated GFR values (all *p* > 0.05).

In the NTN group, renal function parameters were similarly stable over time, with no significant changes in creatinine (*p* = 0.45), cystatin C (*p* = 0.506), measured GFR (*p* = 0.764), or estimated GFR values (all *p* > 0.05).

Notably, serum urea levels decreased significantly in the ON group (55 to 49.5 mg/dL; *p* < 0.001), while no significant change was observed in the NTN group (*p* = 0.319).

### 3.5. Longitudinal Effects and Interaction with Cancer Status

In the T0–T1 repeated-measures analysis, significant main effects of time were observed for serum urea (*p* < 0.001; η^2^ = 0.132), body weight (*p* < 0.001; η^2^ = 0.171), fat mass (*p* = 0.032; η^2^ = 0.045), and arm circumference (*p* = 0.002; η^2^ = 0.113) (Table S2). No significant time effects were observed for renal function parameters, including serum creatinine, cystatin C, and estimated or measured GFR (all *p* > 0.05).

In the same T0–T1 model, no significant time effects were observed for body cell mass, fat-free mass, skeletal muscle index, phase angle, total body water, ECW/ICW ratio, or ECM/BCM ratio. BMI, waist circumference, and hip circumference also did not show significant main effects of time in the repeated-measures model.

In the T0–T1 analysis, no significant time × cancer interaction was detected for any of the evaluated nephrological parameters, body composition indices, or anthropometric measures (all *p* > 0.05), indicating no statistical evidence of differential short-term trajectories between ON and NTN patients (Table S2).

In contrast, in the extended T0–T1–T2 analysis, significant time × cancer interactions were observed for arm circumference (*p* = 0.005; η^2^ = 0.191) and tricipital skinfold thickness (*p* < 0.001; η^2^ = 0.295), indicating different longer-term peripheral anthropometric trajectories according to cancer status (Table S3). No significant time × cancer interactions were observed for renal biomarkers or for most bioimpedance-derived body composition parameters.

## 4. Discussion

The coexistence of chronic kidney disease (CKD) and malignancy requires careful nutritional management, as it involves balancing renal metabolic needs with the prevention of cancer-related malnutrition [1,2,4,5]. Protein intake is central in this setting but remains uncertain. In CKD, protein restriction is recommended to reduce uremic burden and slow disease progression [6,7,8,9,18], whereas oncology guidelines indicate higher protein intake to preserve muscle mass [12,13]. In patients affected by both conditions, these indications are difficult to integrate, and available evidence remains limited. This reflects the lack of studies specifically designed for onconephrological populations, where clinical decisions are often based on indirect data.

The present study evaluated a Mediterranean-style controlled protein diet (MCPD) in patients with CKD and concomitant urological malignancies, compared with patients with CKD alone. The main finding is that a controlled protein intake, including levels below 0.8 g/kg/day in selected patients, was not associated with overt adverse renal or nutritional effects in this retrospective real-world cohort. A consistent reduction in serum urea was observed, while renal function remained stable over time. These findings were broadly comparable between groups for renal biomarkers and most bioimpedance-derived body composition parameters. However, selected peripheral anthropometric measures showed significant time × cancer interactions at extended follow-up, indicating that malignancy may influence specific longer-term body composition trajectories.

From a nephrological perspective, the reduction in serum urea reflects a lower nitrogen load and is consistent with the expected metabolic effects of protein restriction. Urea, however, is not only a marker of renal clearance but also reflects protein metabolism. Elevated urea levels may result from increased protein catabolism, which is commonly observed in conditions of malnutrition and systemic inflammation. In this context, high urea concentrations may indicate metabolic imbalance rather than reduced renal function alone.

In the present cohort, the reduction in urea occurred without any decline in glomerular filtration rate, suggesting that the dietary intervention reduced nitrogen production without inducing a catabolic state. This finding is particularly relevant in onconephrological patients, who are at increased risk of protein–energy wasting due to the combined effects of CKD and cancer-related inflammation. Importantly, the decrease in urea was not associated with deterioration in body composition parameters, supporting the absence of clinically relevant malnutrition.

Previous studies have shown that inflammatory states increase protein turnover and amino acid oxidation, leading to inefficient protein utilization [16,17]. In such conditions, increasing protein intake does not necessarily improve nutritional status and may instead increase nitrogenous waste production. The present findings suggest that a controlled protein intake, when combined with adequate caloric support, may improve metabolic efficiency and reduce unnecessary nitrogen load. This is consistent with evidence showing that dietary quality and energy balance are critical determinants of nutritional status in CKD [9,15].

The Mediterranean dietary pattern may further contribute to these effects through its anti-inflammatory and metabolic properties. Diets rich in plant-based foods, fiber, and unsaturated fats have been associated with reduced systemic inflammation and improved metabolic control [15]. These mechanisms may explain the preservation of lean mass observed in this cohort despite protein restriction.

The stability of renal function is another key finding. Both measured GFR, assessed by iohexol plasma clearance, and estimated GFR remained stable throughout follow-up. This is in line with previous studies showing that low-protein diets may stabilize renal function in CKD [6,7,8]. The absence of decline in GFR, despite protein intake frequently below 0.8 g/kg/day, supports the safety of this approach when nutritional status is maintained.

These results also contribute to the ongoing discussion on optimal protein intake in CKD. Guideline recommendations have evolved over time. Earlier approaches favored more restrictive protein targets, whereas the most recent KDIGO2024 guideline recommends a more moderate intake, with emphasis on individualization and the prevention of protein–energy wasting. In this cohort, protein intake below 0.8 g/kg/day was not associated with adverse outcomes, supporting a flexible approach based on clinical context rather than fixed thresholds.

In oncology, ESPEN guidelines recommend higher protein intake to counteract muscle loss [12,13]. However, these recommendations are based on populations without significant renal impairment. In CKD patients, high protein intake may increase intraglomerular pressure and contribute to renal damage [14]. The present findings suggest that a controlled protein intake, within a balanced dietary framework, may represent an appropriate compromise in onconephrological patients.

Body composition analysis provides further insight into the nutritional trajectories observed during follow-up. In both groups, lean mass parameters were largely preserved, while adiposity-related measures tended to decrease. These findings suggest that, in this structured nephro-nutritional setting, controlled protein prescription was not associated with overt loss of global lean mass when supported by adequate caloric intake [16,17].

Reductions in waist circumference and waist-to-height ratio suggest an improvement in central adiposity, which is associated with cardiovascular risk and CKD progression [11]. These changes may have additional clinical relevance beyond nutritional status. Body composition was assessed using bioelectrical impedance analysis, which allows a more precise evaluation than BMI alone. Phase angle remained stable over time, supporting the preservation of cellular integrity. Reduced phase angle has been associated with worse outcomes in both CKD and cancer populations and represents a useful marker for monitoring nutritional status [16].

Longitudinal analyses showed no significant time × cancer interaction for renal biomarkers and for most bioimpedance-derived body composition parameters. This suggests that, within the limits of this retrospective cohort, onconephrological patients did not show a clearly divergent renal or global nutritional trajectory compared with non-oncological CKD controls under structured nephro-nutritional management. However, in the extended follow-up analysis, arm circumference and tricipital skinfold thickness showed significant time × cancer interactions, with relatively large effect sizes. These findings indicate that cancer status may selectively influence peripheral anthropometric compartments over time. This may reflect differences in regional fat stores, muscle reserve, age-related body composition changes, oncological history, or treatment-related metabolic vulnerability. Therefore, the results should not be interpreted as evidence of identical trajectories between groups, but rather as preservation of renal function and most global body composition parameters, with differential longer-term changes in selected peripheral anthropometric markers.

These findings have relevant clinical implications. A Mediterranean-style controlled protein diet was associated with reduced uremic burden, stable renal function, and preservation of nutritional status in patients with CKD, including those with concomitant malignancy. This supports a tailored approach to nutritional management, in which dietary prescriptions are adapted to renal function, nutritional status, and overall clinical condition.

In this context, a multidisciplinary approach is essential. The management of onconephrological patients requires the integration of nephrological, oncological, and nutritional expertise, as each domain contributes complementary information. Renal function, oncological status, metabolic demands, and nutritional risk must be evaluated together to define an appropriate dietary strategy. This is particularly important when determining protein intake, as both excessive restriction and excessive protein load may negatively affect outcomes.

Some limitations should be considered. First, this is a retrospective single-center study, and the findings should therefore be interpreted as exploratory and hypothesis-generating. Second, dietary adherence was not objectively assessed. Formal measures such as 24 h urinary urea nitrogen, repeated dietary recalls, validated food frequency questionnaires, or nitrogen appearance estimates were not systematically available; therefore, actual protein intake may have differed from the prescribed regimen, and causal attribution to the dietary intervention cannot be established. Third, detailed oncological variables, including tumor stage, remission status, recurrence, systemic treatments, inflammatory burden, and treatment-related metabolic effects, were not consistently available. This limits the interpretation of cancer-specific nutritional trajectories. Fourth, attrition at extended follow-up may have influenced the T0–T1–T2 repeated-measures analyses, particularly for anthropometric parameters showing significant time × cancer interactions. Finally, multiple outcomes were examined, increasing the risk of type I error; therefore, individual *p*-values should be interpreted cautiously and confirmed in prospective studies.

The study also has important strengths. The cohort includes 358 patients, with 183 onconephrological and 175 non-oncological CKD patients, representing a substantial sample size for this clinical setting. A major strength is the systematic use of measured GFR based on iohexol plasma clearance, in addition to estimated GFR. This approach provides a more accurate assessment of renal function, particularly in conditions in which creatinine- or cystatin C-based estimates may be unreliable [9]. In patients with reduced muscle mass, serum creatinine may underestimate renal impairment, while in oncological settings, cystatin C may be influenced by inflammation. The availability of measured GFR, therefore, allowed a more robust evaluation of renal function over time. The integration of detailed body composition analysis further strengthens the assessment of nutritional status.

Urea is the end product of dietary and endogenous protein catabolism and is rapidly excreted in the urine following its synthesis. Its circulating levels are influenced by several non-renal determinants, rendering it a less specific marker of kidney function compared with creatinine. Conditions such as dehydration/hypovolemia and hypercatabolic states are known to increase plasma urea concentrations. Similarly, reductions in plasma volume associated with physical inactivity, or more markedly with prolonged bed rest, together with inadequate protein–energy intake, may contribute to elevated plasma urea levels.

In our study, the observed reduction in serum urea was primarily interpreted in the context of controlled protein intake, which is known to reduce nitrogen load. Importantly, in our cohort, the decrease in fat mass in the presence of preserved lean body mass, as assessed by bioimpedance analysis, does not support the presence of a worsening catabolic state. Rather, this pattern may be indicative of a stable or potentially improved metabolic profile. Therefore, it is plausible that the reduction in serum urea reflects not only decreased dietary protein intake but also improved metabolic efficiency and reduced endogenous nitrogen turnover.

## 5. Conclusions

In conclusion, this retrospective real-world study suggests that structured nephro-nutritional management with an individualized Mediterranean-style controlled protein prescription is feasible in selected patients with CKD and urological malignancies. Renal function and most global bioimpedance-derived body composition parameters were preserved during follow-up, without evidence of overt protein–energy wasting. However, significant time × cancer interactions for arm circumference and tricipital skinfold thickness at extended follow-up suggest that cancer status may influence selected peripheral anthropometric trajectories. These findings support the apparent safety and clinical feasibility of an individualized nephro-nutritional approach, while underscoring the need for prospective studies with objective dietary adherence assessment, detailed oncological characterization, and adequate power to evaluate cancer-specific nutritional outcomes.

## Figures and Tables

**Table 1:** Descriptive statistics were performed for the study cohort, stratified into onconephrological patients (ON) and non-oncological CKD controls (NTN) (a). The distribution of urological malignancies within the ON group is shown in (b). Data are presented as median (range) or number (%). Continuous variables were compared using the Mann–Whitney U test, while categorical variables were analyzed using the χ^2^ test or Fisher’s exact test, as appropriate. *p*-value < 0.05 was considered statistically significant (*).

(a)				
		N (%) Total	Stratified Median (Min–Max)	*p*-Value *
Numbers of patients		358	ON 183 (51.1%)	
			NTN 175 (48.9%)	
Age; median (min-max)		67.0 (18–86)	ON 69 (19–85)	0.031
			NTN 65 (18–86)	
Gender	Male	272 (76%)	ON 152 (83%)	0.002
			NTN 120 (68.6%)	
	Female	86 (24%)	ON 31 (17%)	
			NTN 55 (31.4%)	
BMI; median (min-max)		26.1 (16.7–45.2)	ON 26.05 (16.69–43.21)	0.991
			NTN 26.20 (16.88–45.23)	
BMI Classification	Underweight (<18.5)	5 (1.4)	ON 1 (20)	0.20
			NTN 4 (80)	
	Healthy weight (18.5–24.9)	133 (37.2)	ON 68 (51.1)	
			NTN 65 (48.9)	
	Overweight (25–29.9)	152 (42.6)	ON 84 (55.3)	
			NTN 68 (44.7)	
	Obese (>30)	67 (18.8)	ON 29 (43.4)	
			NTN 38 (56.7)	
Grant Morphotype	Endomorph	159 (44.4)	ON 77 (42.1)	0.487
			NTN 82 (46.9)	
	Mesomorph	114 (31.8)	ON 63 (34)	
			NTN 51 (29.1)	
	Ectomorph	40 (11.2)	ON 19 (10.4)	
			NTN 21 (12.0)	
CKD	Stage II	84 (23.5)	ON 33 (39.3)	0.005
			NTN 51 (60.7)	
	Stage III a	103 (28.8)	ON 56 (54.4)	
			NTN 47 (45.6)	
	Stage III b	102 (28.8)	ON 65 (63.7)	
			NTN 37 (36.3)	
	Stage IV	59 (16.5)	ON 28 (47.5)	
			NTN 31 (52.5)	
	Stage V	4 (1.1)	ON 1 (25)	
			NTN 3 (75)	
Diabetes		54 (15.1)	ON 26 (14.2)	0.660
			NTN 28 (16.0)	
Hypertension (HT)		228 (63.7)	ON 119 (65.0)	0.660
			NTN 109 (62.3)	
Single Kidney		109 (30.45)	ON 86 (47)	0.001
			NTN 23 (13.1)	
**(b)**				
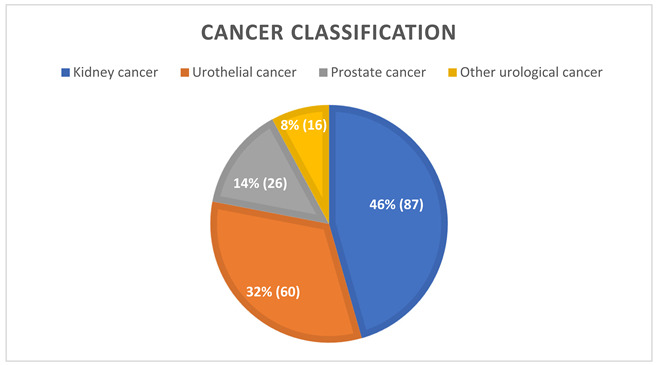				

**Table 3:** Summary of baseline clinical parameters. Estimated GFR (eGFR) was calculated using the CKD-EPI 2021 equation, and measured GFR (mGFR) was obtained using iohexol plasma clearance. Data are presented as median (minimum–maximum). Comparisons between oncological and nephropathic patients were performed using the Mann–Whitney U test. A two-sided *p*-value < 0.05 was considered statistically significant (*).

	Overall Population Median, (Min–Max)	Median, (Min–Max)	*p*-Value
Creatinine (mg/dL)	1.45 (0.64–6.78)	ON 1.54 (0.86–4.47)	0.008 *
		NTN 1.40 (0.68–6.78)	
Cystatin C (mg/L)	1.27 (0.42–5.33)	ON 1.34 (0.67–3.52)	0.027 *
		NTN 1.22 (0.42–5.33)	
mGFR (mL/min/1.73 m^2^)	46.8 (9–126.5)	ON 42.35 (12.04–87)	0.025 *
		NTN 50.70 (9.0–126.5)	
eGFR—SCr (mL/min/1.73 m^2^)	50.78 (7.8–130.09)	ON 48.21 (9.93–104.8)	0.029 *
		NTN 52.48 (7.8–130.09)	
eGFR—SCysC (mL/min/1.73 m^2^)	54.43 (7.87–151.94)	ON 51.04 (12.98–120.36) NTN 56.45 (7.87–151.94)	0.016 *
eGFR SCr-CysC (mL/min/1.73 m^2^)	56.0 (7.6–121.6)	ON 52.96 (11.30–100.83) NTN 58.41 (7.62–121.65)	0.025 *
Urea (mg/dL)	52.00 (17–168)	ON 55.5 (21–153)	0.043 *
		NTN 50 (17–168)	

**Table 4:** Anthropometric indices, body composition, hydration status, and cellular health parameters at baseline (T0) and after 12 months (T1) of dietary intervention. BCM: body cell mass percentage (%); ECM/BCM: extracellular mass to body cell mass ratio; BCMI: body cell mass normalized to height squared (kg/m^2^); BMI: body mass index; ECW/ICW: extracellular water to intracellular water ratio; TBW: total body water percentage (%); FM: fat mass percentage (%); FMI: fat mass normalized to height squared (kg/m^2^); FFM: fat free mass percentage (%); FFMI: fat-free mass normalized to height squared (kg/m^2^); SMI: skeletal muscle mass normalized to height squared (kg/m^2^); PA: phase angle (°). Data are presented as median (minimum–maximum). Longitudinal comparisons between T0 and T1 within each group were performed using the Wilcoxon signed-rank test. A two-sided *p*-value < 0.05 was considered statistically significant. * indicates statistical significance for *p* ≤ 0.05, ** for *p* ≤ 0.01, and *** for *p* ≤ 0.001.

	ON Group Median (Min–Max)		*p*-Value *	NTN Group Median (Min–Max)		*p*-Value
	T0	T1		T0	T1	
BCMI (kg/m^2^)	11.40 (6.72 17.54)	11.54 (6.35–17.18)	0.610	11.48 (6.38–17.08)	10.96 (6.20–15.73)	0.102
BCM (%)	44.45 (25.7–57.7)	44.9 (27.30–55.8)	0.315	44.1 (18.4–57.0)	45.30 (23.20–54.5)	0.491
ECM/BCM	0.85 (0.61–1.29)	0.84 (0.64–1.78)	0.701	0.84 (0.63–1.60)	0.90 (0.65–1.23)	0.831
FMI (kg/m^2^)	4.70 (1.3–18.8)	3.9 (1.4–14.9)	0.014	4.70 (1.4–21.7)	3.6 (1.1–19.4)	0.763
FM (%)	17.60 (7.0–47.7)	16.4 (7.0–42.9)	0.053	17.75 (7.0–54.5)	16.3 (7.0–49.7)	0.232
FFMI (kg/m^2^)	21.20 (14.0–28.6)	21.2 (14.5–28.8)	0.433	21.20 (14.60–35.80)	20.75 (11.8–26.8)	0.256
FFM (%)	82.25 (45.5–93.0)	83.1 (57.2–93.0)	0.500	82.4 (52.6–93.0)	83.7 (50.3–93.0)	0.399
SMI (kg/m^2^)	11.2 (6.2–15.2)	11.2 (6.7–15.0)	0.980	11.1 (6.7–17.2)	10.9 (4.4–14.4)	0.768
PA (°)	6.10 (3.9–8.9)	6.0 (3.6–8.6)	0.566	6.20 (3.3–9.4)	5.70 (3.5–8.7)	0.373
TBW (%)	59.5 (38.0–67.1)	60.30 (41.2–67.1)	0.318	59.35 (32.8–67.1)	61.7 (36.3–67.1)	0.434
ECW/ICW	0.84 (0.57–1.50)	0.83 (0.57–2.16)	0.588	0.84 (0.55–1.96)	0.88 (0.55–1.48)	0.383
Waist circumference (cm)	94 (70–136)	93.0 (74.5–120.0)	0.002	94.5 (64–128)	91 (66.0–113.0)	0.010 **
Hip circumference (cm)	101 (89–139)	100.0 (85–123)	0.922	100 (81–127)	99.0 (84.0–120.0)	<0.001 ***
WHR	0.936 (0.68–1.30)	0.927 (0.49–1.13)	0.014	0.941 (0.69–1.14)	0.932 (0.72–1.1)	0.249
WHtR	0.545 (0.43–1.09)	0.541 (0.42–0.74)	0.002	0.549 (0.39–0.84)	0.532 (0.36–0.70)	0.010 **
BMI (kg/m^2^)	26.05 (16.69–43.21)	25.75 (17.96–36.04)	0.003	26.20 (16.88–45.23)	25.0 (10.38–38.95)	0.008 **

**Table 5:** Longitudinal changes in body composition parameters between T0 and T2 in oncological and nephropathic patients. Data are presented as median (minimum–maximum). Comparisons between T0 and T2 within each group were performed using the Wilcoxon signed-rank test for paired samples. A two-sided *p*-value < 0.05 was considered statistically significant. * indicates statistical significance for *p* ≤ 0.05, ** for *p* ≤ 0.01, and *** for *p* ≤ 0.001.

	ON Group Median (Min–Max)		*p*	NTN Group Median (Min–Max)		*p*
	T0	T2		T0	T2	
BCMI (kg/m^2^)	11.40 (6.72–17.54)	11.73 (7.37–14.04)	0.733	11.48 (6.38–17.08)	10.11 (6.94–15.76)	0.344
BCM (%)	44.45 (25.7–57.7)	46.3 (32.5–55.7)	0.025 *	44.1 (18.4–57.0)	43.3 (24.9–53.8)	0.069
ECM/BCM	0.85 (0.61–1.29)	0.84 (0.67–1.11)	0.239	0.84 (0.63–1.60)	0.96 (0.87–1.20)	0.710
FMI (kg/m^2^)	4.70 (1.3–18.8)	4.60 (1.6–20.7)	0.707	4.70 (1.4–21.7)	3.10 (1.8–18.3)	0.012 *
FM (%)	17.60 (7.0–47.7)	17.15 (7.0–32.5)	0.013 *	17.75 (7.0–54.5)	14.0 (7.1–45.1)	0.018 *
FFMI (kg/m^2^)	21.20 (14.0–28.6)	20.8 (15.5–28.7)	0.537	21.20 (14.60–35.80)	20.2 (15.1–26.7)	0.539
FFM (%)	82.25 (45.5–93.0)	82.8 (67.5–93.0)	<0.001 ***	82.4 (52.6–93.0)	86.0 (54.9–92.9)	<0.001 ***
SMI (kg/m^2^)	11.2 (6.2–15.2)	10.8 (7.2–12.9)	0.232	11.1 (6.7–17.2)	10.8 (6.75–14.2)	0.483
PA (°)	6.10 (3.9–8.9)	6.10 (4.6–7.60)	0.808	6.20 (3.3–9.4)	5.14 (1.19–6.70)	0.480
TBW (%)	59.5 (38.0–67.1)	59.75 (48.7–67.1)	0.014 *	59.35 (32.8–67.1)	62.25 (39.6–67.0)	0.016 *
ECW/ICW	0.84 (0.57–1.50)	0.825 (0.59–1.18)	0.145	0.84 (0.55–1.96)	0.88 (0.74–1.41)	0.566
Waist circumference (cm)	94 (70–136)	92 (73–113)	0.473	94.5 (64–128)	90.5 (67–118)	0.065
Hip circumference (cm)	101 (89–139)	100 (90–119)	0.014 *	100 (81–127)	98 (87–128)	0.359
WHR	0.936 (0.68–1.30)	0.938 (0.68–1.22)	0.293	0.941 (0.69–1.14)	0.884 (0.74–1.02)	0.273
WHtR	0.545 (0.43–1.09)	0.544 (0.45–0.72)	0.434	0.549 (0.39–0.84)	0.527 (0.41–0.71)	0.071

**Table 6:** Nephrological parameters at baseline (T0) and after 12 months (T1) of dietary intervention in nephropathic patients and oncological patients. Data are presented as median (range). Comparisons between T0 and T1 within each group were performed using the Wilcoxon signed-rank test for paired samples. A two-sided *p*-value < 0.05 was considered statistically significant. * indicates statistical significance for *p* ≤ 0.05, ** for *p* ≤ 0.01, and *** for *p* ≤ 0.001.

	ON Group Median (Min–Max)		*p*-Value	NTN Group Median (Min–Max)		*p*-Value
	T0	T1		T0	T1	
Creatinine (mg/dL)	1.54 (0.86–4.47)	1.57 (0.86–3.42)	0.18	1.40 (0.68–6.78)	1.64 (0.80–3.91)	0.57
Cystatin C (mg/L)	1.34 (0.67–3.52)	1.35 (0.74–3.10)	0.43	1.22 (0.42–5.33)	1.36 (0.68–3.63)	0.75
mGFR (mL/min/1.73 m^2^)	42.35 (12.04–87)	41.0 (16.42–78.0)	0.097	50.70 (9.0–126.5)	39.15 (13–67)	0.46
eGFR—SCr (mL/min/1.73 m^2^)	48.21 (9.93–104.8)	46.81 (12.85–103.68)	0.48	52.48 (7.8–130.09)	43.77 (11.16–79.22)	0.33
eGFR—SCysC (mL/min/1.73 m^2^)	51.04 (12.98–120.36)	50.43 (15.97–114.08)	0.28	56.45 (7.87–151.94)	49.52 (12.85–112.69)	0.57
eGFR SCr-CysC (mL/min/1.73 m^2^)	52.96 (11.30–100.83)	49.92 (15.11–101.99)	0.813	58.41 (7.62–121.65)	50.12 (13.02–92.72)	0.825
Urea (mg/dL)	55 (21–153)	49.0 (26–123)	<0.001 ***	50 (17–168)	47 (17–103)	0.01 *
Urea > 50 (mg/dL)	65 (50–153)	52 (29–123)	<0.001 ***	68 (50–168)	50 (32–103)	<0.001 ***

## Data Availability

The data presented in this study are available on request from the corresponding author due to privacy concerns.

## Supplementary Materials

The following supporting information can be downloaded at: https://www.mdpi.com/article/10.3390/nu18121863/s1; Table S1. Nephrological parameters at baseline (T0) and second follow-up (T2) in nefropatic patients and oncological patients. Comparisons between T0 and T2 within each group were performed using the Wilcoxon signed-rank test for paired samples. A two-sided *p*-value < 0.05 was considered statistically significant. * indicates statistical significance for *p* ≤ 0.05, ** for *p* ≤ 0.01, *** for *p* ≤ 0.001. Table S2. Repeated-measures ANOVA (T0–T1) and time × cancer interaction analysis. Data are presented as p-values and partial η^2^ derived from a mixed-design repeated-measures ANOVA. A two-sided *p*-value < 0.05 was considered statistically significant. Table S3. Repeated-Measures ANOVA (T0–T1-T2) and Time×Cancer Interaction (mettere in supplementary senza commentarla).
