# Supplementary material for: Rethinking Onconephrology: A Nephro-Nutritional Integrated Approach in Patients with Chronic Kidney Disease and Urological Malignancies
Source: Nutrients. 2026 Jun 9;18(12):1863. doi: 10.3390/nu18121863 (PMC13305153; doi:10.3390/nu18121863)
Supplement: Supplementary file 1 [file nutrients-18-01863-s001.zip › nutrients-4277223-supplementary.pdf]

Supplementary Materials:

Table S1. Nephrological parameters at baseline (T0) and second follow-up (T2) in nefropatic patients and oncological patients. Comparisons between T0 and T2 within each group were performed using the Wilcoxon signed-rank test for paired samples. A two-sided p-value <0.05 was considered statistically significant.

* indicates statistical significance for p ≤ 0.05, ** for p ≤ 0.01, *** for p ≤ 0.001.

	**ON group** Median (min-max)		**p**	**NTN group** Median (min-max)		**p**
	T0	T2		T0	T2	
**Creatinine (mg/dL)**	1.54 (0.86–4.47)	1.73 (0.91-2.76)	0.676	1.40 (0.68–6.78)	1.54 (0.91-2.76)	0.45
**Cystatin C (mg/L)**	1.34 (0.67–3.52)	1.46 (0.64-3.22)	0.540	1.22 (0.42–5.33)	1.21 (1.06-2.33)	0.506
**mGFR (mL/min/1.73m²)**	42.35 (12.04–87)	37.9 (13.7-76.0)	0.519	50.70 (9.0–126.5)	41.0 (27-62)	0.764
**eGFR - SCr (mL/min/1.73m²)**	48.21 (9.93-104.8)	42.29 (15.33-101.38)	0.720	52.48 (7.8-130.09)	44.41 (25.48-78.77)	0.764
**eGFR - SCysC (mL/min/1.73m²)**	51.04 (12.98-120.36)	45.28 (16.28-118.78)	0.604	56.45 (7.87-151.94)	59.16 (25.29-66.27)	0.511
**eGFR SCr-CysC (mL/min/1.73m²)**	52.96 (11.30–100.83)	47.50 (15.55–105.39)	0.428	58.41 (7.62–121.65)	52.44 (25.27–69.79)	0.515
**Urea (mg/dL)**	55 (21–153)	49.5 (22-108)	**<0.001*** **	50 (17–168)	44 (24-55)	0.319

3.5 Longitudinal effects and interaction with cancer status

Repeated-measures analysis of variance demonstrated significant time effects for several variables (Table S2). In particular, serum urea decreased over time ($p<0.001$; $\eta^2=0.132$), as did body weight ($p<0.001$; $\eta^2=0.171$) and fat mass ($p=0.032$; $\eta^2=0.045$). Arm circumference also showed a significant reduction over time ($p=0.002$; $\eta^2=0.113$). No significant time effects were observed for renal function parameters, including serum creatinine, cystatin C, and estimated or measured GFR (all $p>0.05$).

With respect to body composition, no significant time effects were observed for body cell mass (BCM), fat-free mass (FFM), skeletal muscle index, phase angle, total body water, ECW/ICW ratio, or ECM/BCM ratio (all $p>0.05$). Similarly, anthropometric measures such as BMI, waist circumference, and hip circumference did not show significant changes over time in the repeated-measures model.

No significant time × cancer interaction was detected for any of the variables analyzed, including nephrological parameters, body composition indices, and anthropometric measures (all $p>0.05$). This indicates that temporal changes were comparable between onconephrological (ON) and non-oncological CKD (NTN) patients.

Table S2. Repeated-measures ANOVA (T0–T1) and time × cancer interaction analysis. Data are presented as p-values and partial η² derived from a mixed-design repeated-measures ANOVA. A two-sided p-value <0.05 was considered statistically significant.

Variable	p(Time)	η²(Time)	p(Time×Cancer)	η²(Interaction)
**Creatinine**	.672	.001	.241	.009
**Cystatin C**	.488	.003	.861	.000
**eGFR (M)**	.138	.014	.588	.002
**Urea**	**<.001**	**.132 (scende il valore del tempo)**	**.229**	**.013**
**Weight**	<.001	.171	.229	.004
**BMI**	.692	.001	.250	.012
**BCM**	.784	.001	.272	.012
**BCMI**	.076	.031	.053	.037
**FM**	.032	.045	.664	.002
**FMI**	.369	.008	.889	.000
**FFM**	.383	.008	.169	.019
**FFMI**	.369	.008	.889	.000
**SMI (kg/ m²)**	.125	.023	.227	.014
**PA (Phase Angle)**	.080	.030	.072	.032
**TBW**	.558	.003	.212	.015
**ECW/ICW**	.369	.008	.889	.000
**ECMB/BCM**	.902	.000	.685	.002
**Waist**	.412	.007	.328	.010
**Ombelical**	.074	.034	.430	.007
**Hips**	.412	.007	.328	.010
**Arm circumference**	**.002**	**.113 (grande effetto)**	**.190**	**.010**
**Tricipital skinfold**	.775	.001	.142	.031

Table S3. Repeated-Measures ANOVA (T0–T1-T2) and Time×Cancer Interaction (mettere in supplementary senza commentarla)

Variable	p(Time)	η^2(Time)	p(Time x Cancer)	η^2(Time x Cancer)
**Creatinine**	0,695	0,004	0,889	0,001
**Cystatin C**	0.45	0.013	0.77	0.004
**eGFR (M)**	0,711	0,005	0,834	0,002
**Urea**	**0,431**	**0,016**	**0,067**	**0,055**
**Weight**	**0,079**	**0,067**	**0,599**	**0,011**
**BMI**	**0,073**	**0,071**	**0,521**	**0,014**
**BCMI**	**0,627**	**0,012**	**0,801**	**0,005**
**BCM**	**0,027**	**0,093**	**0,989**	**0,000**
**FMI**	**0,669**	**0,006**	**0,612**	**0,008**
**FM**	**0,012**	**0,116**	**0,827**	**0,005**
**FFMI**	**0,045**	**0,081**	**0,105**	**0,059**
**FFM**	**0,067**	**0,074**	**0,960**	**0,001**
**SMI**	**0,189**	**0,046**	**0,146**	**0,054**
**PA**	**0,187**	**0,045**	**0,132**	**0,056**
**TBW**	**0,071**	**0,074**	**0,927**	**0,002**
**ECW/ICW**	**0,288**	**0,034**	**0,918**	**0,002**
**Waist**	**0,065**	**0,097**	**0,560**	**0,017**
**Ombelical**	**0,426**	**0,029**	**0,895**	**0,004**
**Hips**	**0,695**	**0,006**	**0,520**	**0,015**
**Arm**	**0,003**	**0,214**	**0,005**	**0,191**
**Tric. skinfold**	**0,000**	**0,309**	**0,000**	**0,295**
**ECMB/BCM**	**0,085**	**0,185**		

Repeated-measures ANOVA across three time points (T1–T3) showed significant time effects for fat mass (P=0.012), arm circumference (P=0.003), and tricipital skinfold thickness (P<0.001). Significant time–by–cancer interactions were observed for arm circumference (P=0.005) and tricipital skinfold (P<0.001), indicating different temporal patterns between participants with and without cancer. No significant interactions were observed for renal biomarkers or other body composition parameters.

Data are presented as p values, and partial η^2 obtained from a repeated-measures analysis of variance (mixed ANOVA).
